# Diagnostic Value of Bone SPECT/CT in Patients with Suspected Osteomyelitis

**DOI:** 10.4274/mirt.galenos.2019.20053

**Published:** 2019-09-06

**Authors:** Pelin Arıcan, Berna Okudan, Rıza Şefizade, Seniha Naldöken

**Affiliations:** 1 University of Health Sciences, Ankara Numune Training and Research Hospital, Clinic of Nuclear Medicine, Ankara, Turkey

**Keywords:** Bone scintigraphy, osteomyelitis, infection, SPECT, SPECT/CT

## Abstract

**Objectives:**

The aim of our retrospective study was to evaluate the contribution of single photon emission computed tomography/computed tomography (SPECT/CT) to three phase bone scintigraphy/SPECT for the assessment of osteomyelitis (OM) and patient’s management.

**Methods:**

Eighty-five patients who were suspected as having OM were included in this study. Tc-99m MDP three phase bone scintigraphy and SPECT/CT were performed to the region of suspected OM. SPECT/CT findings were compared with the findings of planar images/SPECT. Both planar bone scan/SPECT and SPECT/CT findings were divided into two groups: With OM and without OM. In all patients, scintigraphic diagnosis was confirmed by clinical follow up, laboratory findings, microscopic-bacteriological examinations, radiological, surgical, and pathological findings.

**Results:**

SPECT/CT changed the diagnosis and treatment planning in 14/85 (16.5%) patients. SPECT/CT was significantly superior to planar scan/SPECT imaging for determining OM (kappa value was 0.626 for planar scan/SPECT, 0.929 for SPECT/CT). SPECT/CT was statistically more successful in detection of chronic OM, and useful in differentiating chronic OM from acute OM (kappa value was 0.541 for planar scan/SPECT, 0.944 for SPECT/CT).

**Conclusion:**

SPECT/CT increases accuracy of the diagnosis in the evaluation of OM when it is compared to three phase bone scintigraphy/SPECT. SPECT/CT can change the diagnosis and management of the patients.

## Abstract (tr)

**Amaç:**

Retrospektif çalışmamızda osteomiyelitin (OM) değerlendirilmesinde ve hasta yönetiminde tek foton emisyon bilgisayarlı tomografi/ bilgisayarlı tomografi (SPECT/BT) üç fazlı kemik sintigrafisi/SPECT’ye katkısı araştırıldı.

**Yöntem:**

OM’den şüphelenilen 85 hasta çalışmaya dahil edildi. OM şüphesi bulunan bölgeye Tc-99m MDP üç fazlı kemik sintigrafisi ve SPECT/BT yapıldı. SPECT/BT bulguları planar görüntü/SPECT bulguları ile karşılaştırıldı. Planar kemik sintigrafisi/SPECT ve SPECT/BT bulguları iki gruba ayrıldı: OM olmayanlar ve OM olanlar. Tüm hastalarda sintigrafik tanı, klinik takip, laboratuvar, mikroskobik-bakteriyolojik inceleme, radyoloji, cerrahi ve patoloji ile doğrulanmıştır.

**Bulgular:**

SPECT/BT, 14/85 (%16,5) hastada tanı ve tedavi planlamasını değiştirdi. SPECT/BT, OM’nin belirlenmesinde planar görüntüler/SPECT görüntülemeye göre anlamlı derecede üstün bulundu (kappa değeri planar tarama/SPECT için 0,626, SPECT/BT için 0,929). SPECT/BT, kronik OM’nin saptanmasında istatistiksel olarak daha başarılıydı ve akut OM’den kronik OM’nin ayırt edilmesinde faydalı idi (kappa değeri planar tarama/SPECT için 0,541, SPECT/BT için 0,944).

**Sonuç:**

SPECT/BT, üç fazlı kemik sintigrafisi/SPECT ile karşılaştırıldığında OM değerlendirilmesinde tanı doğruluğunu arttırmaktadır. SPECT/BT hastaların teşhis ve yönetimini değiştirebilir.

## Introduction

Osteomyelitis (OM) is a bone infection. Early diagnosis of OM is essential for a successful therapy and management of the complications. Determining whether the OM is acute or chronic is very important for deciding an appropriate antimicrobial and surgical treatment. The diagnosis of bone infection is still challenging. Clinical and laboratory parameters such as erythrocyte sedimentation rate (ESR), C-reactive protein (CRP), microbiological examination are usually insufficient to make a correct diagnosis (1,2,3). The changes in plain radiography occur late and are non-specific. Computed tomography (CT) and magnetic resonance imaging (MRI) are able to show pathologic morphological changes in the bones and surrounding soft tissues. Although they are very sensitive, their specificity is moderate (3). Three-phase planar bone scintigraphy (3-phase PBS) is the first option among radionuclide techniques for detection of the OM since it is widely available, easy, and inexpensive. This technique has a high sensitivity, but its specificity is limited (1,2,3,4). PBS must be combined with single photon emission computed tomography (SPECT) to obtain high sensitivity and specificity however, their value is limited due to the poor accuracy in localizing the increased uptake (4,5). SPECT/CT can improve the prognostic value of planar radionuclide techniques since it evaluates morphologic and functional information together (4,5). Use of SPECT/CT significantly increases the diagnostic accuracy of skeletal scintigraphy (6). Nowadays, SPECT/CT has been used frequently in orthopedic diseases (7,8,9,10). In this retrospectively study, we aimed to evaluate the contribution and superiority of SPECT/CT to 3-phase PBS/SPECT for assessment of OM and management of patients.

## Materials and Methods

### Patients

We retrospectively analyzed 85 patients who were suspected as having OM with clinical and laboratory findings and we performed the 3-phase PBS-SPECT/CT. The mean age of the patients was 50±32 years (range 18-82 years). There was clinical suspicion of bone infection (pain, swelling, erythema, heat, fever, wound etc.), and abnormal laboratory findings (increased number of white blood cells and neutrophils, ESR and CRP) during 10-90 days in the selected patients. Patients without follow up, patients with prostheses or metallic instrumentation causing artifacts on CT, patients who had an operation within 3 months and using antibiotic therapy for more than 7 days were excluded. The study group was consisted of 34 females and 51 males. There were systemic diseases in 22 patients (diabetes mellitus n=16, tuberculosis n=2, ankylosing spondylitis n=2, others=2). All patients had X-rays before radionuclide imaging and clinical follow up for at least 6 months. The patients’ characteristics are summarized in Table 1. All patients gave their written informed consents for the 3-phase PBS and SPECT/CT study. Local Ethics Committee approved the present retrospective study (3739/0.01.2014).

### Three-phase PBS

After a bolus injection of the 740 MBq technetium-99m methylene diphosphonate (Tc-99m MDP) (Monrol, Eczacıbaşı, Turkey), perfusion images were obtained immediately by acquiring blood flow images, 1 frame for 60 sec, at 64x64 matrix. Blood pool images were acquired after perfusion (anterior-posterior position, 256x256 matrix, 500,000-750,000 counts). Whole body and static images were obtained 3 hours after the injection (whole body scan 8 cm/min, static images 500,000-750,000 counts). The images were acquired with a dual head gamma camera, equipped with a low energy, high resolution, large-field-of-view parallel-hole collimator (Millennium Hawkeye 4, GE Medical Systems, Milwaukee, WI).

### SPECT/CT

After the 3-phase PBS, SPECT/CT was performed on the region of suspected OM in all patients, firstly, CT scan was obtained. Secondly, SPECT scan was taken at the same time in the supine position. Low dose CT scan acquisition parameters were 140 kV voltage, 2.5 mA tube current, 512x512 pixel matrix, 5 mm slice thickness, and 3 mm reconstruction. SPECT acquisition parameters were 128x128 pixel matrix, 360° acquisition, 6° steps and 25 sec per frame. SPECT data was reconstructed according to the ordered subset expectation maximization iterative technique.

### Image interpretation

Images were interpreted by two qualified nuclear medicine specialists who were blinded to all clinical and radiological details of the patients (p<0.005). But they had information about the suspicious infection area due to the dynamic study. Each nuclear medicine specialist independently looked at the first 3-phase PBS and SPECT images together using a linear grey scale display. The uptake of perfusion, blood pool and delayed phase were compared with the opposite side. Then, SPECT/CT fusion images were read at the same time using color display. On the basis of the findings on the 3-phase PBS/SPECT and SPECT/CT, patients were divided into two groups: with OM, without OM (no OM).

**Without OM:** Normal radiotracer uptake on perfusion-blood pool phase, normal, slightly or mild increased radiotracer uptake in the delayed phase with no abnormal morphologic changes or detection of osteoarthritis-degenerative-traumatic-postoperative changes, osteonecrosis, heterotopic ossification on CT were described as no OM.

**With OM:** Intense or mild diffuse or focal increased radiotracer uptake at the lesion site in all three phases in 3-phase PBS was defined OM. Detection of periosteal reaction, small focus of gas or foreign bodies, soft tissue abscesses and edema on CT with scintigraphic findings were considered as acute OM (AOM). Bone destruction, sequestration, involucra, fistulous tract on CT associated with the radiotracer uptake were interpreted as chronic OM (COM).

### Final Diagnosis

All patients were followed up in our hospital for 6 months. The final diagnosis was verified by microbiologic examination in 39 patients, by radiology and scintigraphic techniques in 31 patients (CT=8, MRI=17, gallium=6), and by surgery and histopathologic findings in 15 patients. We could not use the same gold standard, because there were not microbiologic-histopathologic results in all patients.

### Statistical Analysis

Kappa test was used to compare 3-phase PBS/SPECT and SPECT/CT. Sensitivity, specificity, positive predictive value (PPV), negative predictive value (NPV), and accuracy of each method were calculated.

## Results

Three-phase PBS/SPECT found OM in 48 (AOM=30, COM=18) (56.4%) patients. There was no OM in 37 (43.6%) patients. While OM was determined in 44 (AOM=16, COM=28) (51.7%) patients with SPECT/CT, it was not determined in 41 (48.3%) patients. In final diagnosis, OM was found in 49 (AOM=21, COM=28) (57.6%) patients. There was no OM in 36 patients. The results of 3-phase PBS/SPECT, SPECT/CT and final diagnosis are given in detail in the Table 2 and 3.

When the findings of 3-phase PBS/SPECT and SPECT/CT were compared with the findings of the final diagnosis, it was observed that 3-phase PBS/SPECT predicted the correct diagnosis in 69 (82.1%) patients when SPECT/CT predicted the correct diagnosis in 83 (97.6%) patients. SPECT/CT changed the diagnosis and the treatment in 14 of 85 (16.5%) patients. Three-phase PBS/ SPECT showed false positive results in 11, and false negative results in 5 patients. SPECT/CT gave false positive results in 2 patients. There was no false negative result in SPECT/CT. The false positive and false negative results and the contribution of SPECT/CT are seen in the Table 4.

Sensitivity, specificity, PPV, NPV and accuracy of each method were calculated. The results are showed in the Table 5. When the kappa values of 3-phase PBS/SPECT and SPECT/CT were analyzed together, it was seen that SPECT/CT was significantly superior to PBS/SPECT in imaging for determining OM (kappa value was 0.626 for planar scan/SPECT, 0.929 for SPECT/CT). In addition, SPECT/CT was statistically more successful in detection of COM, and useful in differentiating COM from AOM (kappa value was 0.541 for PBS/SPECT, 0.944 for SPECT/CT).

## Discussion

The diagnosis of OM is challenging. The localization of the infection site and determination of acute or COM are important for planning the treatment. The treatment of OM is multidisciplinary. It is very important to take the appropriate antimicrobial treatment and to choose the patients for surgery. Untreated or insufficiently treated bone infection leads to destruction and recurrence of diseases. Three-phase PBS is widely used for evaluation of the suspected OM (1,2,3,4). SPECT/CT has become an increasingly important diagnostic modality in addition to 3-phase PBS in orthopedic diseases (7,8,9,10). In this study, we investigated the contribution and superiority of SPECT/CT to 3-phase PBS/SPECT in the patients who were suspected as having OM and underwent 3-phase PBS.

Three-phase PBS in the diagnosis of OM has high sensitivity, but its specificity is limited (1,2,3). SPECT/CT increases sensitivity and specificity in diagnosis of OM (4,5,7,8). We found that SPECT/CT was a more sensitive and specific method in comparison to PBS/SPECT for diagnosing OM (sensitivity 88.3% versus 100%; specificity 75.6% versus 95.4%). SPECT/CT improved specificity rather than sensitivity. Analysis of the kappa values showed that SPECT/CT was significantly better than PBS/SPECT in the diagnosis of OM. Horger et al. (7) found that sensitivity was 78% for PBS/SPECT and SPECT/CT. But specificity of SPECT/CT was higher than PBS/SPECT. It was 50% for PBS/SPECT and 86% for SPECT/CT. In our experiment, sensitivity was similar with this study, but specificity was significantly higher for both techniques. In our study, the number of patients who had a history of fracture and several bone surgery was more than in the study of Horger et al. (7).

**Figure 1:**
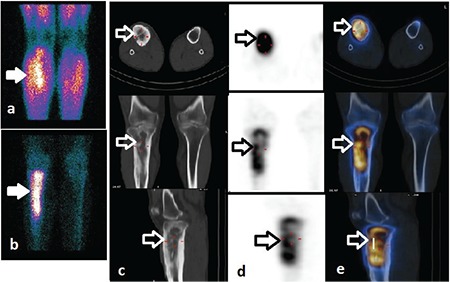
Three-phase planar bone scintigraphy of a 41-year-old man with right leg pain and erythema. (A) Blood pool (B) late static images show hyperemia and increased osteoblastic activity in right upper half tibia (arrows). (C) Axial, (D) coronal (E) sagittal computed tomography (CT), single photon emission computed tomography (SPECT) and SPECT/CT images. The heterogeneous increased uptake and chronic morphological changes are seen in the fusion images (arrows). The planar images suggest acute osteomyelitis without SPECT/CT. But chronic osteomyelitis is described with the morphological changes in the CT images. Chronic osteomyelitis is confirmed by pathology

In our study, SPECT/CT showed actual anatomical localization of radiotracer uptake and allowed true localization, extension, and activation of infection. Actual anatomic localization was particularly useful for differentiating soft tissue and bone infection. Morphologic changes on CT also helped to make a correct diagnosis. While soft tissue infection was detected in only 5 patients with 3-phase PBS/SPECT, 11 patients were interpreted with soft tissue infection with SPECT/CT in our study. The most important contribution of SPECT/CT was the change of the diagnosis and treatment in 14 of 85 (16.5%) patients. SPECT/CT changed the diagnosis as true positive in five patients who had false negative results with PBS/SPECT. In 9 of 11 patients who had false positive results with PBS/SPECT, accurate diagnosis was provided by SPECT/CT. It had no contribution to diagnosis in two patients. Osteoarthritis was found with CT in one patient who was reported to have COM with SPECT/CT. She was suffering from active rheumatoid arthritis and had chronic morphologic changes on CT. Therefore, the findings of bone scan and SPECT/CT should be interpreted with clinical information. In the other patient who was reported to have AOM with SPECT/CT, the final diagnosis was postoperative changes which was supported with clinic follow up and microbiologic results. This patient had had surgery five months before the scan. Both techniques failed to differentiate postoperative changes from infection. Three-phase PBS could be interpreted as false positive in this period, because bone surgery leads to increase in bone turnover and osteoblastic activity. Accurate differentiation of AOM from COM is very important for patient management. Increased radiotracer uptake is found on all three phases of 3-phase PBS in both AOM and reactive COM. As AOM is treated with antimicrobial therapy, surgery with antimicrobial therapy may be needed in COM (11). SPECT/CT is important in differentiating AOM from reactive COM. In our experience, SPECT/CT correctly classified COM in 8 of 36 patients who were interpreted as AOM on 3-phase PBS (Figure 1). We saw that SPECT/CT was particularly useful for the diagnosis of post-traumatic and post-operative OM in the patients with reactive COM.

**Figure 2:**
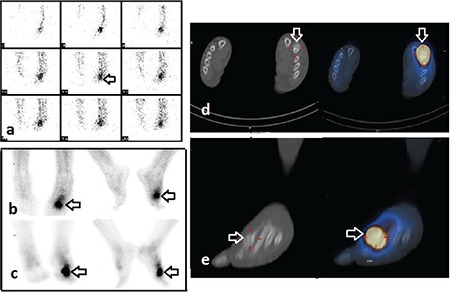
Three-phase PBS of a 56-year-old woman with left foot swelling, pain, and trauma history for 2 years. (A) Perfusion (B) blood pool (C) late static images show slightly increased perfusion, blood pool and osteoblastic activity in left metatarsophalangeal region (arrows). The planar images suggest acute osteomyelitis without single photon emission computed tomography (SPECT)/computed tomography (CT). (D) Axial (E) sagittal CT, and fusion images show fracture site and callus in the distal of second metatarsal bone associated with focal intense increased uptake (arrows). Fracture and callus are confirmed by diagnostic CT

**Figure 3:**
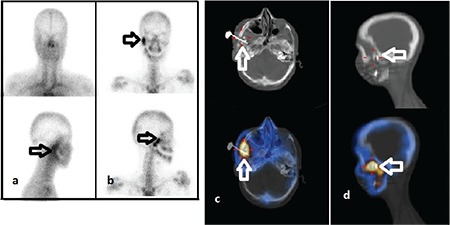
A 18-year-old woman who was operated due to hemifacial atrophy and was being suspected as having osteomyelitis in right mandibula five months after the operation. (A) There is mild hyperemia on the right temporomandibular region in blood pool (B) delayed anterior and right lateral static images show intense focal increased radiotracer uptake in the same area with hyperemia (arrows). (C) Axial and (D) sagittal single photon emission computed tomography/ computed tomography, images show intense focal increased radiotracer uptake around the metal implant on the zygomatic bone (arrows). These findings are interpreted as acute osteomyelitis. The result of microbiological examination is reported as infection

**Figure 4:**
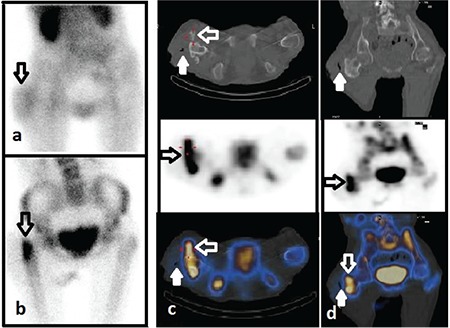
A 22-year-old woman who had right hemiplegia and was suspected as having osteomyelitis in the right upper femur. (A) Blood pool anterior image shows heterogeneous hyperemia in the soft tissues just lateral to the trochanter major (arrows). (B) The intense osteoblastic uptake is seen on trochanteric regions in the anterior late static image. (C) Axial, (D) coronal single photon emission computed tomography (SPECT)/CT images show fistula tract in the soft tissue (white arrows). Fusion images show that radiotracer uptake around the trochanter major is associated with the bone structures within soft tissue (arrows). The findings of SPECT/computed tomography (CT) are described as heterotopic ossification and soft tissue infection. This diagnosis is confirmed by CT and pathology

SPECT/CT improved the image quality, resolution and identified minimal increased activity. SPECT/CT might easily evaluate the small multiple osseous structure in hand-wrist, and foot-ankle (12,13). SPECT/CT confirmed final diagnosis in 40 out of 41 patients with suspected lesions in their hands and feet (Figure 2). It is sometimes difficult to identify an infection focus in the appendicular skeleton such as skull, vertebra or pelvis with 3-phase PBS, because of compact bone structure and superposition of bones (14). In our study, there were three patients with suspected OM of skull after operation. OM sites could be diagnosed accurately in these patients by SPECT/CT (Figure 3). Bolouri et al. (15) performed bone SPECT/CT to evaluate patients who were suspected as having OM on the jaw. They found that SPECT/CT slightly improved the specificity of 3-phase PBS. Damle et al. (16) reported a case with skull base OM. Planar image was equivocal, but SPECT/CT was helpful in the detection of OM. Spinal OM and spondylodiscitis are localized in vertebral body and intervertebral disc. Soft tissue abscesses often accompany spinal infection (1). Since detailed anatomic localization is possible, SPECT/CT is helpful in differentiating OM from spondylodiscitis. Additionally, fracture and degenerative changes can be differentiated from infection with CT component. SPECT/CT confirmed the final diagnosis in 5 patients with spine lesions in our study. We found COM in 1 patient, soft tissue infection in 1 patient, fracture in 1 patient, degenerative change in 2 patients. Distinguishing insufficiency or traumatic fractures from infection in pelvic region is very important. In this study, there were 2 patients who were suspected as having OM in pelvis. SPECT/CT correctly diagnosed soft tissue infection in one patient and revealed heterotopic ossification and soft tissue infection in the other patient who was suspected as having OM (Figure 4). The therapy of those patients completely changed. Linke et al. (8) reported that bone SPECT/CT findings led to change in the diagnosis of one third of patients with pain in the extremities (17). The highest rate of diagnostic alteration was found 60% among patients who were suspected as having OM (8).

### Study Limitations

There were three limitations in our study. The main limitation was the retrospective design of the study. Despite the fact that the nuclear medicine specialists did not know the clinical and radiological results of the patients, they had information about the region of suspected OM due to perfusion and blood pool images.

The second limitation was that there was lack of standard gold reference for the final diagnosis of OM. All patients did not have microbiologic and histopathologic examinations. We had to compare the 3-phase PBS and SPECT/CT results with different references. Because of this reason, there was no standardization in the final diagnosis.

The third limitation in our study was that poor image quality due to low dose CT. Even though low dose CT was enough for anatomic localization of radiotracer uptake; the morphologic evaluation of particularly small and compact bone structures in the foot, hand, mandibula, and spine was difficult.

## Conclusion

Three-phase PBS combined with SPECT/CT is an useful tool in diagnosis of OM. We think that SPECT/CT has more diagnostic accuracy than 3-phase PBS/SPECT in differentiation of reactive COM and AOM. Especially SPECT/CT is superior in postoperative and posttraumatic OM. SPECT/CT is very successful in the evaluation of skull, vertebra, pelvis, spine, hand, and foot than PBS. We recommend that SPECT/CT should be used in selected patients.

## Figures and Tables

**Table 1:**
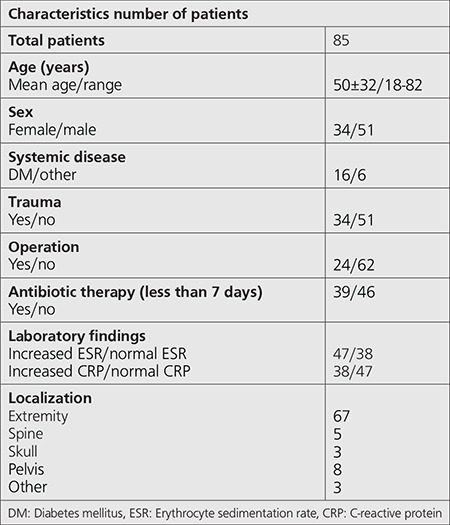
Patients’ characteristics

**Table 2:**
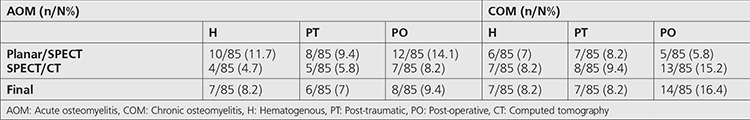
The results of planar scan/single photon emission computed tomography (SPECT), SPECT/CT, and final diagnosis in patients without osteomyelitis

**Table 3:**
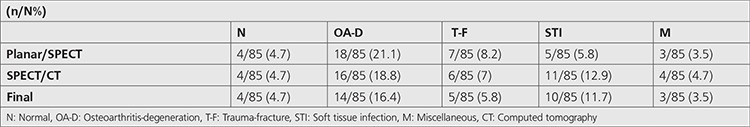
The results of planar scan/single photon emission computed tomography (SPECT), SPECT/CT, and final diagnosis in patients without osteomyelitis

**Table 4:**
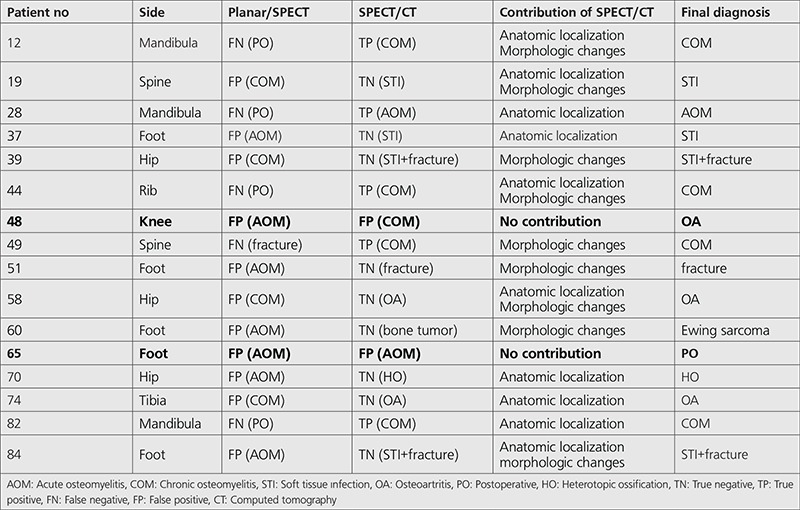
The contribution of single photon emission computed tomography (SPECT)/CT in the patients who had false negative and false positive results

**Table 5:**
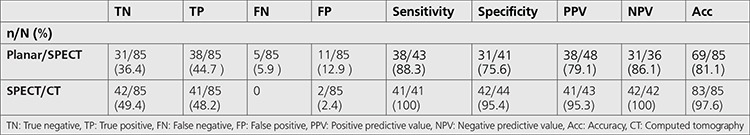
Planar scan/single photon emission computed tomography (SPECT) and SPECT/CT assessment of osteomyelitis

## References

[1] Palestro CJ (2015). Radionuclide imaging of osteomyelitis. Semin Nucl Med.

[2] Bruggen van der W, Bleeker-Rovers CP, Boerman OC, Gotthardt M, Oyen WJ (2010). PET and SPECT in osteomyelitis and prosthetic bone and joint infections: a systematic review. Semin Nucl Med.

[3] Gotthardt M, Bleeker-Rovers CP, Boerman OC, Oyen WJ (2010). Imaging of inflammation by PET, conventional scintigraphy, and other imaging techniques. J Nucl Med.

[4] Huellner MW, Strobel K (2014). Clinical applications of SPECT/CT in imaging the extremities. Eur J Nucl Med Mol Imaging.

[5] O’Connor MK, Kemp BJ (2006). Single photon emission computed tomography/ computed tomography: basic instrumentation and innovations. Semin Nucl Med.

[6] Mariani G, Bruselli L, Kuwert T, Kim EE, Flotats A, Israel O, Dondi M, Watanabe N (2010). A rewiew on the clinical uses of SPECT/CT. Eur J Nucl Med Mol Imaging.

[7] Horger M, Eschmann SM, Pfannenberg C, Storek D, Vonthein R, Claussen CD, Bares R (2007). Added value of SPECT/CT in patients suspected of having bone infection: preliminary results. Arch Orthop Trauma Surg.

[8] Linke R, Kuwert T, Uder M, Forst R, Wuest W (2010). W. Skeletal SPECT/CT of the peripheral extremities.. AJR Am J Roentgenol.

[9] Even-Sapir E, Flusser G, Lerman H, Lievshitz G, Metser U (2007). SPECT/multislice low-dose CT: a clinically relevant constituent in the imaging algoritm of nononcologic patients referred for bone scintigraphy. J Nucl Med.

[10] Arıcan P, OkudanTekin B, Şefizade R, Naldöken S, Bastuğ A, Özkurt B (2015). The role of bone SPECT/CT in the evaluation of painful joint prostheses. Nucl Med Commun.

[11] Wing VW, Jeffrey RB Jr, Federle MP, Helms CA, Traton P (1985). Chronic osteomyelitis examined by CT. Radiology.

[12] Schleich FS, Schürch M, Huellner MW, Hug U, von Wartburg U, Strobel K, Veit-Haibachn P (2012). Diagnostic and therapeutic impact of SPECT/CT in patients with unspecific pain of the hand and wrist. EJNMMI Res.

[13] Singh VK, Javed S, Parthipun A, Sott AH (2013). The diagnostic value of single photon-emission computed tomography bone scans combined with CT (SPECT-CT) in diseases of the foot and ankle. Foot Ankle Surg.

[14] Younis JA (2018). Additive value of 99mTechnetium methylene diphosphonate hybrid single-photon emission computed tomography/computed tomography in the diagnosis of skull base osteomyelitis in otitis externa patients compared to planar bone scintigraphy. World J Nucl Med.

[15] Bolouri C, Merwald M, Huellner MW, Velt-Haibach P, Kuttenberger J, Perez-Lago M, Seifert B, Strobel K (2013). Performance of orthopantomography planar scintigraphy, CT alone, and SPECT/CT in patients with suspected osteomyelitis of the jaw. Eur J Nucl Med Mol Imaging.

[16] Damle NA, Kumar R, Kumar P, Jaganathan S, Patnecha M, Bal C, Bandopadhyaya G, Malhotra A (2011). SPECT/CT in the diagnosis of skull base osteomyelitis. Nucl Med Mol Imaging.

[17] Govaert GA, IJpma FF, McNally M, McNally E, Reininga IH, Glaudemans AW (2017). Accuracy of diagnostic imaging modalities for peripheral posttraumatic osteomyelitis - a systematic review of the recent literature. Eur J Nucl Med Mol Imaging.

